# Cholecystokinin (CCK) Is a Mediator Between Nutritional Intake and Gonadal Development in Teleosts

**DOI:** 10.3390/cells14020078

**Published:** 2025-01-08

**Authors:** Hangyu Li, Hongwei Liang, Xiaowen Gao, Xiangtong Zeng, Shuo Zheng, Linlin Wang, Faming Yuan, Shaohua Xu, Zhan Yin, Guangfu Hu

**Affiliations:** 1 Hubei Hongshan Laboratory, College of Fisheries, Huazhong Agricultural University, Wuhan 430070, Chinam13605305830@163.com (X.Z.);; 2 Key Lab of Freshwater Biodiversity Conservation Ministry of Agriculture, Yangtze River Fisheries Research Institute, The Chinese Academy of Fisheries Sciences, Wuhan 430223, China; 3 State Key Laboratory of Freshwater Ecology and Biotechnology, Institute of Hydrobiology, Chinese Academy of Sciences, Wuhan 430072, China

**Keywords:** satiety neuropeptide, follicle-stimulating hormone, GnRH3, pituitary, sexual maturity

## Abstract

Nutritional intake is closely linked to gonadal development, although the mechanisms by which food intake affects gonadal development are not fully understood. Cholecystokinin (CCK) is a satiety neuropeptide derived from the hypothalamus, and the present study observed that hypothalamic CCK expression is significantly influenced by food intake, which is mediated through blood glucose levels. Interestingly, CCK and its receptors were observed to exhibit a high expression in the hypothalamus–pituitary–gonad (HPG) axis of grass carp (*Ctenopharyngodon idellus*), suggesting that CCK is potentially involved in regulating fish reproduction through the HPG axis. Further investigations revealed that CCK could significantly stimulate the expression of gonadotropin-releasing hormone-3 (GnRH3) in the hypothalamus. In addition, single-cell RNA sequencing showed that *cckrb* was highly enriched in pituitary follicle-stimulating hormone (FSH) cells. Further study confirmed that CCK can significantly induce FSH synthesis and secretion in primary cultured pituitary cells. Additionally, with primary cultured ovary cells as a model, the in vitro experiment demonstrated that CCK directly induces the expression of *lhr*, *fshr*, and *cyp19a1a* mRNA. This indicates that hypothalamic CCK may act as a nutrient sensor involved in regulating gonadal development in teleosts.

## 1. Introduction

Nutritional intake is closely linked to gonadal development. In mammals, it has been observed that over nutrition can trigger precocious sexual maturation in rats and monkeys [1,2,3], while inadequate nutrition tends to delay sexual maturation [4,5]. In teleosts, food intake is closely linked to the age of sexual maturity. Food restriction delays both growth and sexual maturity [6]. These results emphasize how dietary intake levels affect fish gonadal development. However, the precise mechanisms by which food intake modulates sexual maturation are still not fully understood.

In mammals, cholecystokinin (CCK) is a satiety neuropeptide, and food intake could significantly upregulate CCK expression in the hypothalamus [7,8,9,10]. Similarly, in fish, feeding has been shown to have the ability to increase hypothalamic CCK transcription levels [11,12]. However, the physiological mechanisms underlying diet-induced hypothalamic CCK expression in fish remain unclear. Recent studies in mammals have identified CCK-expressing neurons as glucose-sensing neurons capable of detecting changes in blood glucose concentration [13,14]. This has raised the question of whether fish CCK neurons are also glucose sensitive and whether feeding induces hypothalamic CCK expression by raising the blood glucose levels. At present, these questions remain unanswered.

In the mammalian hypothalamus, CCK is recognized as a post-prandial satiety factor, providing short-term satiety and inhibiting appetite to maintain a balance between food intake and weight regulation [15,16]. The regulation of feeding behavior and the secretion of gonadotropin-releasing hormone (GnRH) are closely linked, with various anorexic and anorexigenic signals controlling feeding behavior and altering GnRH secretion via receptors on GnRH neurons. This relationship has been studied in mice, with GT1-7, a mouse hypothalamic neuronal cell line, being commonly used as an in vitro model to investigate the expression of feeding-related peptide receptors and their roles in GnRH secretion [17]. Beyond its role in regulating feeding, CCK is proposed to be involved in reproductive control by stimulating the migration and activation of gonadotropin-releasing hormone (GnRH) neurons in the mammalian brain [18,19]. In goldfish (*Carassius auratus*), feeding has been shown to increase hypothalamic CCK transcript levels [20], and exogenous CCK injections can inhibit fish feeding in grass carp (*Ctenopharyngodon idellus*) and Siberian sturgeon (*Acipenser baerii Brandt*) [21,22], suggesting that CCK may also act as an appetite suppressant in fish. In addition, recent research has identified the highly specific expression of CCK receptors (CCKRs) in the pituitary follicle-stimulating hormone (FSH)-secreting cells of tilapia (*Oreochromis mossambicus*) [18], and CCK has been demonstrated to stimulate gonadotropin secretion in the pituitary gland of goldfish [19]. The results indicate that CCK could play a role in regulating sexual maturation in fish by mediating pituitary gonadotropins. Based on the above information, we propose that feeding can modulate gonadal development through the mediation of the satiety factor CCK in fish.

In order to test this hypothesis, this study focused on the grass carp as a model for research. Firstly, both in vivo and in vitro experiments were conducted to investigate the relationship between CCK and key reproductive factors (GnRH, gonadotropins, and sex steroid hormones) within the hypothalamic–pituitary–gonadal axis, providing further insights into the regulatory mechanisms of sexual maturation by CCK in fish. Secondly, we demonstrated the mechanism of feeding-induced CCK expression and identified the key regulators of CCK expression in the fish hypothalamus. This provided valuable insights into the mechanisms by which CCK induces sexual maturation in fish. Building on these findings, our ultimate goal was to gain a deeper understanding of how feeding modulates gonadal development via the satiety factor CCK.

## 2. Materials and Methods

### 2.1. Animals and Drugs

One-year-old (1+) grass carp weighing between 1.0 and 2.5 kg were obtained from the fish farm of Huazhong Agricultural University, China. The fish were housed in 250-L tanks at a temperature of 22 ± 1 °C and maintained on a 12 h light:12 h dark photoperiod for seven days. The animals were provided with a commercial grass carp diet from Tongwei (Chengdu, China) at a rate of 3% of their body weight twice daily over the course of the study. Subsequently, the females were subjected to screening using primers that were specific to the sex of the subject (P_F_: AGCAGCAGGTAGCGGAAGAG; P_R_: AATAACGACAGTTGACAGGATTGAATG) [23]. The use of grass carp in this study was conducted in accordance with the guidelines of the Animal Care and Use Committee of Huazhong Agricultural University (Ethical Approval No. HBAC20091138; date: 15 November 2009). The grass carp CCK1 (DYLGWMDF-NH2) and CCK2 (DYVGWMDF-NH2) peptides were produced by GenScript Corporation (Piscataway, NJ, USA), and their respective sequences are shown in Figure S1A. Dimethyl sulfoxide (DMSO) was employed as the solvent for these peptides. Further details on the administered drugs can be found in Table S1.

### 2.2. Cell Transfection with Dual Luciferase Assay

In order to characterize the functional coupling of carp CCKRs with ligand selectivity, the functional expression of CCKRs was carried out in HEK-293 cells (Catalog No. WN-10257, Warner Bio, Wuhan, China) in accordance with our recent study [24]. Firstly, the open reading frame (ORF) of grass carp CCKRs were sub-cloned into the eukaryotic expression vector pcDNA3.1/Zeo(-) to generate the expression vector of the CCKRs (pcDNA3.1/Zeo(-)-CCKRs). Subsequently, the pcDNA3.1/Zeo(-)-CCKRs were transfected into HEK-293 cells to establish stable cell lines expressing CCKRs. Following this, the CRE-Luc reporter, NFAT-Luc reporter, and green fluorescent protein (GFP) reporter (serving as an internal control) were transiently transfected into HEK-293 cells with the stable expression of grass carp CCKRa (GeneID:127516394), CCKRb (GeneID:127519351), and CCKRl (GeneID:127509240), respectively. Subsequently, the HEK-293 cells were treated with various CCK peptides. Following a 24 h period of drug treatment, the cells were lysed using a passive lysis buffer (E1941, Promega, Beijing, China). Lysate samples of the cells were analyzed for firefly luciferase activities using a Dual-Glo^TM^ Luciferase Assay Kit (E2920, Promega, Beijing, China) in a Lumat LB9507 luminometer (EG&G, Gaithburg, MD, USA).

### 2.3. Cell Culture

The hypothalamus and pituitaries of grass carp were isolated and processed using the trypsin/DNase II/EDTA digestion protocol [25], while the ovaries were treated with the collagenase II/DNase I/EDTA method [26]. The dissociated cells from the brain, pituitary, and ovary were seeded into 24-well plates at a density of 1 × 10^5^ cells/mL per well. Initial culturing was performed in plating medium at 28 °C in a 5% CO_2_ environment. Following 24 h of cultivation, the cells were exposed to specific drugs for another 24 h.

### 2.4. Immunofluorescence

The hypothalamus and pituitaries of grass carp were dissected and fixed in 4% paraformaldehyde in phosphate-buffered saline (PBS) for 24 h. All samples were subsequently embedded in paraffin wax and sectioned at a thickness of 5 μm. In the immunofluorescence experiment, the paraffin-embedded tissue sections were subjected to a series of dewaxing and rehydration procedures. Subsequently, the tissue section was immersed in citric acid antigen repair buffer and subjected to microwave irradiation at a high to medium temperature to facilitate antigen retrieval. For the purpose of immunofluorescence staining, the slides were incubated for 30 min in 1×PBST (PBS containing 0.1% Tween-20) supplemented with 5% normal goat serum. The slides were then incubated at 4 °C in a humidified chamber overnight with primary antibodies. Further details regarding the antibodies are provided in Table S2. Following three washes with 1×PBST, the secondary antibodies were applied for one hour at 37 °C in a humidified chamber. Both primary and secondary antibodies were appropriately diluted in 1×PBST supplemented with 5% normal goat serum. After incubation with the secondary antibody, the slides were washed three to five times in 1×PBST and some of the samples were labelled with 4′,6-diamidino-2-phenylindole (DAPI). The visualization and observation of signals were conducted using either a FV1000 confocal microscope (Olympus, Tokyo, Japan) or a Leica TCS SP8 microscope (Leica Microsystems, Wetzlar, Germany).

### 2.5. Single-Cell RNA-Seq Library Construction and Sequencing

The tissues were washed in ice-cold minimum essential medium and dissociated using trypsin, ethylenediamine tetraacetic acid (EDTA), and DNase II. After cell counting, the cells were washed twice in NeuroGro medium (T710KJ, BasalMedia, Shanghai, China) and then resuspended at a concentration of 1 × 10^6^ cells per ml in PBS. The resuspended cells were then added to the well of the SeekOne^®^ DD Chip S3 (Chip S3). For the hypothalamus and pituitary samples, 12 and 10k cells were loaded onto the chip, respectively. Subsequently, barcoded hydrogel beads (BHBs) and partitioning oil were added separately to their respective wells within Chip S3. After the emulsion droplets had been generated, reverse transcription was performed at a temperature of 42 °C for 90 min, followed by inactivation at 85 °C for 5 min. The cDNA was extracted from the disrupted droplets and amplified by PCR. The amplified cDNA product was subjected to a series of steps including purification, fragmentation, end repair, A-tailing, and ligation to sequencing adapters. Indexed PCR was subsequently conducted to amplify the DNA, representing the 3′ polyA region of expressed genes including the cell barcodes and unique molecular index. The indexed sequencing libraries were purified using SPRI beads and quantified by quantitative PCR (KAPA Biosystems KK4824, Wilmington, MA, USA).

The libraries were then sequenced using either an Illumina NovaSeq 6000 platform (San Diego, CA, USA) with PE150 read length or a DNBSEQ-T7 platform with PE150 read length. Subsequently, the raw sequencing data were processed by Fastp, which involved trimming the primer sequence and eliminating low-quality bases. Subsequently, the SeekOneTools software (https://seeksoul.online) was employed to preprocess the sequencing data and align it to the GRCm38 reference genome to obtain a gene expression matrix. For further analysis of the scRNA-Seq data, the R packages Seurat (version 4.3.0.1)71 and Harmony (version 0.1.1)72 were employed for filtering, data normalization, dimensionality reduction, clustering and further analysis of the single-cell RNA-Seq data. The marker genes are presented in Tables S3 and S4.

### 2.6. Real-Time PCR

Total RNA was extracted using the MolPure^®^ Cell/Tissue Total RNA Kit (Yeasen Biotech, Shanghai, China) following the provided protocol. The purified RNA was then reverse-transcribed into cDNA using the HiScript III 1st Strand cDNA Synthesis Kit (+gDNA wiper) (Vazyme, Cat#R312, Nanjing, Jiangsu, China). Quantitative RT–PCR (RT–qPCR) was conducted using the ChamQ Universal SYBR qPCR Master Mix (Vazyme, Cat# Q711) on a Quantagene q225. The relative gene expression levels were normalized to *β-actin*. All experiments were conducted in triplicate on at least three separate occasions. The primer sequences are provided in Supplementary Table S5.

### 2.7. Measurement of FSH Secretion and Glucose

To investigate the regulatory effects of CCK on the secretion of FSH in the grass carp pituitaries, we obtained pituitaries from 15 to 18 immature grass carp. Primary cultures of grass carp (2-year-old) pituitary cells were prepared by the trypsin digestion method [23]. The pituitary cells were then seeded in 24-well cell culture plates at a density of 2.5 × 10^6^ cells per well and incubated with CCK1 and CCK2. After drug treatment, the cell culture medium was collected for hormone secretion analysis. The quantification of grass carp FSH protein secretion was conducted utilizing an ELISA assay kit produced by our laboratory (Patent No. CN202211185349.8). Fluorescence signals were quantified using a FluoStar OPTIMA fluorescence plate reader (BMG Labtech GmbH, Ortenberg, Germany). In the feeding experiment, sixteen healthy grass carp were selected and temporarily reared in a 250 L tank. Prior to the feeding experiment, the fish were fed once daily for seven consecutive days. Blood samples were collected at 0 h (before feeding) and at 1, 3, and 6 h post-feeding. Blood glucose levels were measured using a blood glucose monitor (Accu-Chek Performa, Roche, Basel, Switzerland).

### 2.8. Intraperitoneal Injection of CCK in Grass Carp

Prior to the commencement of the experiments, the female grass carp, with a weight range of 1.0 to 1.5 kg, were anesthetized with 0.05% MS222 in accordance with the animal regulations of Huazhong Agricultural University. The stock solution of CCK peptide dissolved in DMSO was dissolved in fish saline and 200 μg of mature peptide was injected per kg of fish body weight. Subsequently, the CCK peptides were administered by intraperitoneal injection to eight grass carps in each of the two experimental groups. Concurrently, eight control fish received an equivalent volume of fish saline containing the same concentration of DMSO. Grass carp were anesthetized for gonad removal 24 h after injection and then placed in cryopreservation tubes and stored in a liquid nitrogen tank. Following sample collection, the total RNA was extracted for reverse transcription, and RT-qPCR was used to detect the relevant gene expression changes.

### 2.9. Data Transformation and Statistical Analysis

Transcript levels were measured via qRT-PCR using ABI 7500 SDS v1.5.1 software, with calibration achieved through standard curves (dynamic range: 10^5^, correlation coefficient: >0.95). β-Actin served as the internal control, and the target gene mRNA levels were normalized and expressed as a percentage of the mean value (“% Ctrl”). Data, pooled from four to eight replicates (mean ± SEM), were analyzed using one-way ANOVA to identify significant differences among treatments. Dunnett’s post hoc test was conducted in SPSS Statistics 26.0 (Chicago, IL, USA). Significance was denoted as *p* < 0.05 (“*”) or *p* < 0.01 (“**”), while different letters represented significant differences (*p* < 0.05) between groups.

## 3. Results

### 3.1. Analysis of CCK and Its Activation of the Receptor

**Figure 1:**
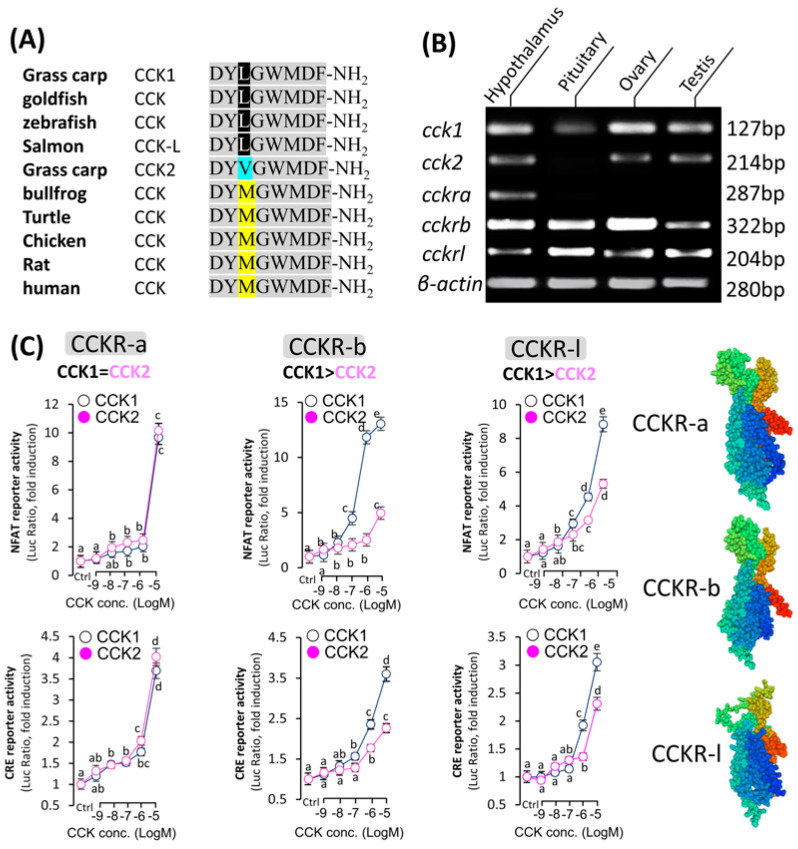
Analysis of the aptitude of CCK and its receptor. (**A**) Amino acid sequence alignment of mature peptides among grass carp CCK1 and CCK2. (**B**) Tissue expression profile of carp CCKs and CCKRs. (**C**) Ligand selectivity of grass carp CCK-Rs for CCKs. To verify the affinity of grass carp CCKRa, CCKRb, and CCKRl to the two ligands (CCK1 and CCK2), the NFAT-Luc and CRE-Luc reporter genes were co-transfected with GFP into HEK293T, which can stably express grass carp CCKRa, CCKRb, and CCKRl. Then, the cells were treated with CCK1 or CCK2 for 24 h. Statistical significance is represented by different letters at *p* < 0.05.

In grass carp, two subtypes of CCK have been successfully cloned and identified as CCK1 and CCK2, respectively. Sequence analysis revealed that each subtype encodes a mature peptide consisting of exactly eight amino acids (Figure S1A). Sequence alignment across different species revealed a difference of only one amino acid in the mature CCK peptide sequence (Figure 1A), indicating the conservation of CCK and its potential involvement in important functions. Phylogenetic analysis based on nucleotide sequence further confirmed that the newly cloned CCK cDNAs could be clustered in the evolutionary branch of fish CCKs. It was observed that CCK1 and CCK2 were split into two separate evolutionary branches, but both were similar to CCK1 in zebrafish (Figure S1B). In addition, three CCK receptor subtypes, namely CCKRa, CCKRb, and CCKRL, were also identified in grass carp. Co-transfection of HEK-293 cells expressing CCKRa, CCKRb, and CCKRl with CCK1 and CCK2 all resulted in the expression of NFAT-Luc and CRE-Luc luciferase activities in a dose-dependent manner (Figure 1C). CCK1 demonstrated a higher activation efficiency for CCKRb and CCKRl compared to CCK2; however, CCK1 exhibited a similar activation rate to CCK2 in the CCKRa activation assay. After confirming the sequences of grass carp *cck* and its receptor *cckr*, we detected their transcript levels in the grass carp hypothalamus–pituitary–gonad axis. The study found that *cck1* was expressed in the hypothalamus, pituitary, and gonads of grass carp, while *cckra* was not detected in the pituitary. Additionally, both *cckrb* and *cckrl* were highly expressed in the grass carp HPG axis (Figure 1B and Figure S1C). This evidence suggests that the CCK/CCKR system may play a significant role in the hypothalamus–pituitary–gonad axis of grass carp.

### 3.2. Food Intake Induces CCK Expression in the Hypothalamus

**Figure 2:**
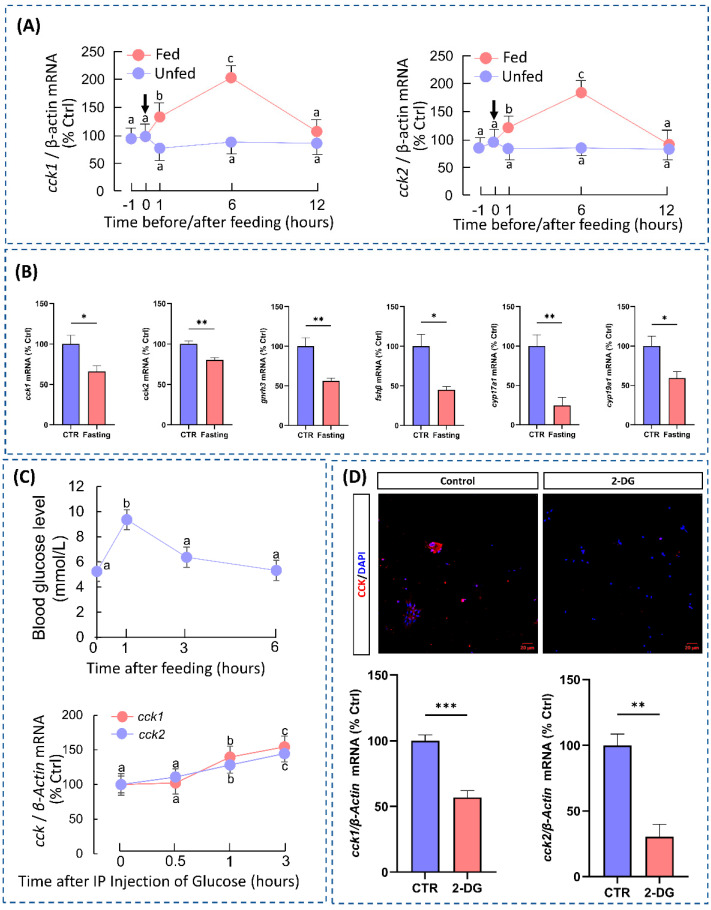
Food intake induces CCK expression in the hypothalamus. (**A**) Postprandial changes in grass carp hypothalamus *cck1* and *cck2* mRNA expression were observed within 12 h. (**B**) Changes in reproduction-related hormones were observed in grass carp after a 7 day period of starvation stress. (**C**) Changes in blood glucose concentration were observed in postprandial grass carp as well as changes in CCK transcript levels in the hypothalamus after glucose injection. (**D**) Changes in cck in the hypothalamic cells of grass carp after 24 h of 2-DG treatment (40×). Different letters mean significant differences. * *p* < 0.05. ** *p* < 0.01. *** *p* < 0.001.

**Figure 3:**
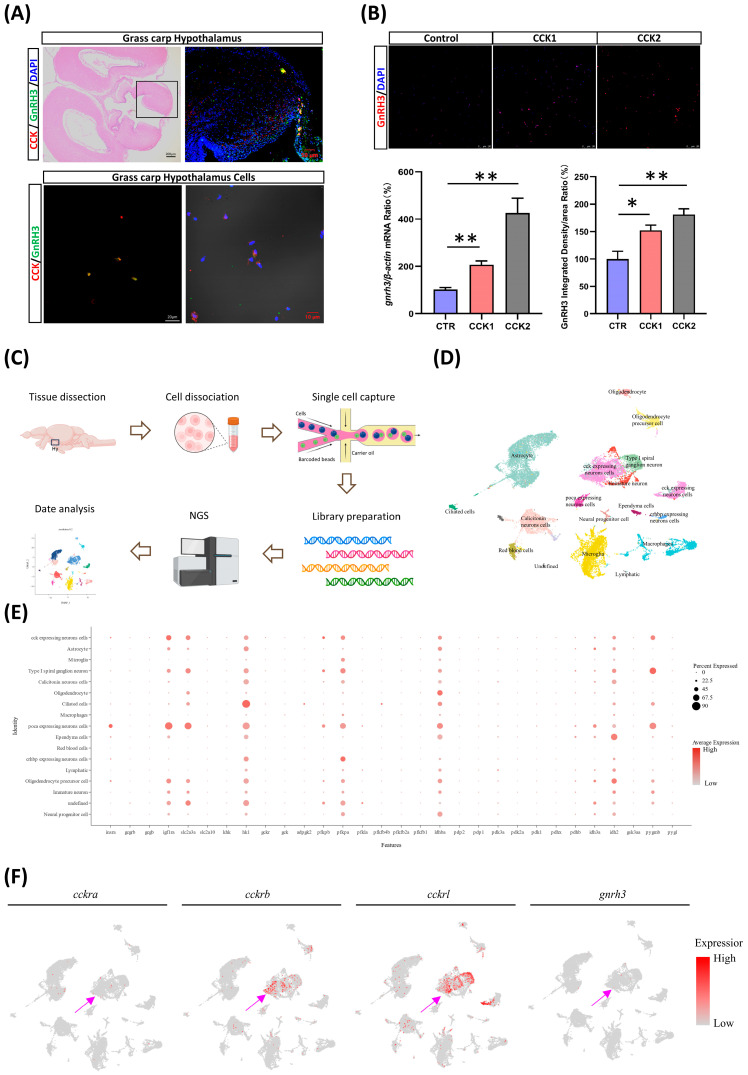
CCK induces the expression of GnRH3 in the hypothalamus. (**A**) Immunohistochemistry showing co-localization of CCK (red) and GnRH3 (green) in the hypothalamic tissues and cells. Black box shows immunohistochemistry area. (**B**) CCK1 and CCK2 induced GnRH3 (red) expression in the grass carp hypothalamic cells. * *p* < 0.05. ** *p* < 0.01. (**C**) Workflow of single-cell RNA-Seq of grass carp hypothalamus. (**D**) UMAP visualization shows the unsupervised clustering of the aggregate of scRNA-Seq experiments, revealing 17 major clusters of hypothalamic cells present in grass carp. (**E**) The dot plot displaying the percentage and average expression of genes related to glucose metabolism. (**F**) The UMAP visualization of *cckra*, *cckrb*, *cckrl*, and *gnrh3*. Purple arrow indicates area of expression.

There was a significant increase in the expression levels of *cck1* and *cck2* mRNA in the hypothalamus of grass carp 6 h after feeding (Figure 2A). Conversely, after one week of fasting, there was a significant decrease in *cck1* and *cck2* mRNA expression in the hypothalamus. It is interesting to note that starvation markedly suppressed *gnrh3* expression in the hypothalamus, *fshβ* in the pituitary, and *cyp17a1* and *cyp19a1* in the ovaries (Figure 2B). To explain the mechanism behind feeding-induced CCK expression, we found that the blood glucose levels were significantly elevated 1 h after feeding by measuring the blood glucose levels in grass carp at different times after feeding. Based on this finding, we intraperitoneally injected glucose into the grass carp and found that the expression levels of hypothalamic cck1 and cck2 were elevated after 1 h (Figure 2C). In vitro, we found that 2-DG significantly inhibited CCK expression in the hypothalamic cells (Figure 2D). Moreover, the analysis of the single-cell transcriptome revealed that glucose-sensing genes were highly expressed in the CCK-expressing neurons (Figure 3E). These results indicate that CCK acts as a satiety factor in grass carp, and feeding may trigger an upregulation of hypothalamic CCK expression by elevating the blood glucose levels.

### 3.3. CCK Stimulates the Expression of GnRH3 in the Hypothalamus

The analysis of tissue expression showed that CCK and its receptor are highly expressed in the hypothalamus of grass carp (Figure S1C). Subsequently, immunohistochemistry was used to detect the CCK distribution in the hypothalamus of grass carp using a CCK antibody. The results showed that CCK was mainly distributed in the arcuate nucleus region of the hypothalamus, which is also the main distribution region of GnRH3. Dual fluorescence immunolocalization analysis demonstrated the co-expression of CCK and GnRH3 in the same brain cells (Figure 3A). To characterize cellular heterogeneity in the hypothalamus, we performed scRNA-Seq on cells dissociated from the hypothalamus of grass carp (Figure 3C). Seurat was used to identify cell clusters and Uniform Manifold Approximation and Projection (UMAP) was used for visualization (Figure 3D). In addition, the single-cell transcriptome analysis showed that *cckrb* and *cckrl* were detected in GnRH3-expressing neurons (Figure 3F). The results suggest that CCK may regulate GnRH3 expression in the hypothalamus through autocrine or paracrine mechanisms. To test the hypothesis, the in vitro experiment found that CCK could significantly induce GnRH3 mRNA expression and synthesis in grass carp hypothalamic cells (Figure 3B). Meanwhile, the analysis of single-cell transcriptome results revealed that glucose metabolism-related genes were also found to be highly expressed in the CCK-overexpressing neurons (Figure 3E).

### 3.4. CCK Could Induce FSH Synthesis and Secretion in the Pituitary

**Figure 4:**
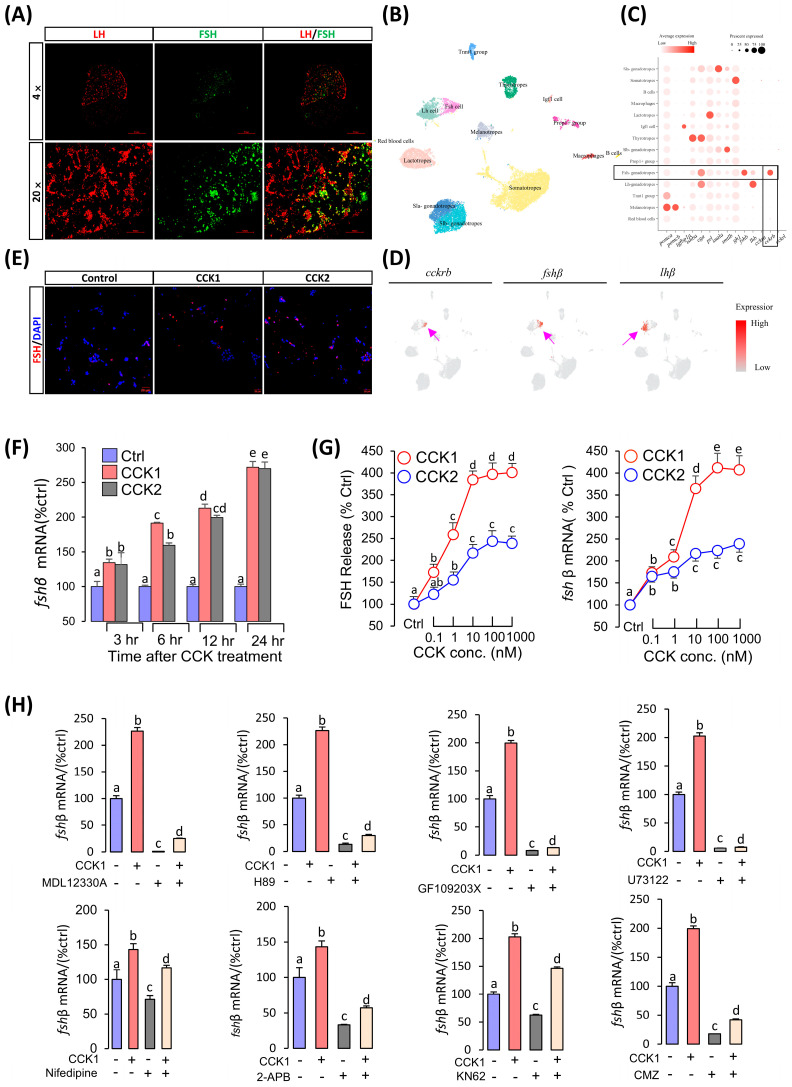
CCK induces FSH synthesis and secretion in the pituitary. (**A**) Immunofluorescence localization analysis of LH (red) and FSH (green) in the pituitary gland. (**B**) The UMAP visualization displays the unsupervised clustering of the aggregate of scRNA-Seq experiments, revealing 14 major clusters of pituitary cells. (**C**) Dot plot demonstrating the percentage and mean expression of the most prevalent genes in the pituitary gland. (**D**) UMAP visualization of *cckra*, *cckrb*, *cckrl*, and *gnrh3*. Purple arrow indicates area of expression. (**E**) Immunohistochemical analysis of CCK induced-FSH levels in pituitary cells (40×). (**F**) Time-dependent experiments of CCK1 and CCK2 on *fshβ* mRNA expression in the grass carp pituitary cells. (**G**) Dose-dependent experiments of CCK1 and CCK2 on the regulation of FSH protein secretion and *fshβ* mRNA expression in the grass carp pituitary cells. (**H**) Signal transduction of CCKs-regulated *fshβ* mRNA expression in the grass carp pituitary cells. Different letters mean significant differences.

In contrast to mammals, immunofluorescence showed that grass carp luteinizing hormone (LH) and FSH were secreted from two different cells (Figure 4A). Single-cell transcriptome analysis revealed that CCKRb was only highly expressed in grass carp pituitary FSH cells, but very low in the LH cells (Figure 4C,D). These findings suggest that CCK can directly affect pituitary FSH cells. To examine the pituitary actions of CCK, carp CCK1 and CCK2 were synthesized and tested in the primary culture of carp pituitary cells. In our initial study, 24 h 100 nM static incubation with CCK1 or CCK2 was able to elevate FSH synthesis (Figure 4E). Time-course experiments also revealed that CCK1 and CCK2 could significantly stimulate *fshβ* mRNA expression from 1 µM to 3 in 24 h (Figure 4F). In dose-dependence studies, 24 h incubation with increasing levels of CCK1 or CCK2 (0.1–1000 nM) also triggered FSH release and *fshβ* mRNA expression in a dose-related fashion (Figure 4F). To further elucidate the signal transduction for FSH regulation by CCK1 and CCK2, the possible involvement of the AC/cAMP/PKA, PLC/PKC, and Ca^2+^/CaM pathways was examined. As shown in Figure 4H, co-treatment with AC inhibitor MDL12330A, PKA inhibitor H89, PLC inhibitor U73122, VSCC blocker nifedipine, PKC inhibitor GF109203X, IP3 receptor blocker 2-APB, CaM-II inhibitor KN62, or CaM antagonist CMZ could all block the stimulatory effects of CCK1 (1 μM) on *fshβ* mRNA expression. The results suggest that CCK-induced *fshβ* mRNA expression was caused by AC/cAMP/PKA, PLC/PKC, and Ca^2+^/CaM activation.

### 3.5. CCK Could Induce the Sexual Steroid Hormone Synthesis in the Gonad

**Figure 5:**
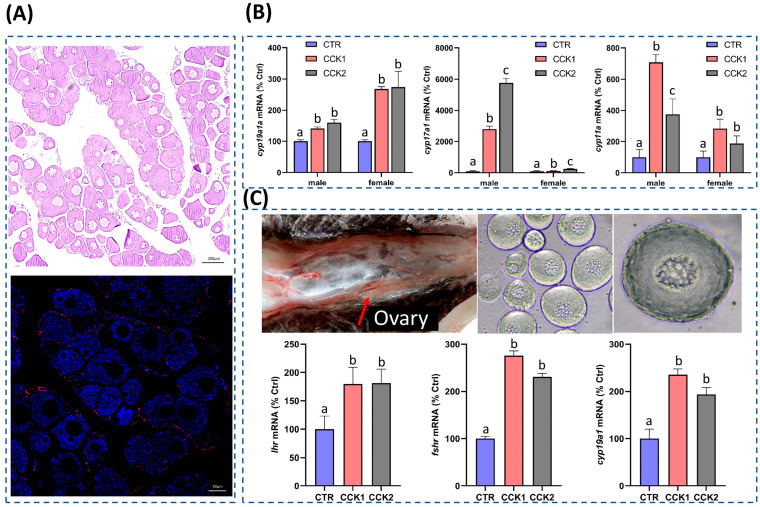
Functional studies of CCK in the grass carp gonads. (**A**) Immunofluorescence localization of CCK in the ovary. (**B**) Effect of CCK on *cyp11a*, *cyp17a1*, and *cyp19a1a* mRNA expression in the female and male gonads. (**C**) Effect of CCK on *lhr*, *fshr*, and *cyp19a1a* mRNA expression in oocytes. Different letters mean significant differences.

Tissue expression analysis revealed the high expression of *cck* and its receptor *cckrb* and *cckrl* in the grass carp ovary and testes (Figure 1B). Immunofluorescence localization analysis demonstrated that the ovary secreted the CCK maturation peptide (Figure 5A). Intraperitoneal injection of CCK1 and CCK2 could significantly induce the expression of the sexual steroid hormone synthetase genes including *cyp11a*, *cyp17a1*, and *cyp19a1a* (Figure 5B). Using primary cultured ovary cells as a model, it was found that CCK1 was able to significantly induce the mRNA expression of *fshr*, *lhr*, and *cyp19a1a* (Figure 5C). The above results indicate that CCK can directly act on the ovary to promote the synthesis of ovarian sexual steroid hormones.

## 4. Discussion

Previous studies have shown that CCK is a neuropeptide that induces satiety and is secreted in the hypothalamus in mammals. Food intake significantly increases CCK expression in the hypothalamus [7,8,9,10,27]. Recent research in mammals has revealed a fascinating connection between neurons that express CCK and glucose sensing [13,14]. In addition, the study observed the expression genes related to glucose metabolism in hypothalamic CCK-expressing neurons. These neurons are capable of detecting changes in blood glucose concentrations, indicating a potential connection between blood glucose regulation and CCK. In this study, which was conducted on grass carp, we revealed similarities between the regulation of blood glucose levels and hypothalamic CCK expression in response to food intake. These compelling results suggest that glucose may be a key factor in inducing CCK expression in the hypothalamus of grass carp, and that food intake stimulates hypothalamic CCK expression by elevating the blood glucose levels. Furthermore, it has been demonstrated that a deficiency in CCK is associated with gonadal abortion in zebrafish and medaka [28,29]. Given the role of CCK in regulating satiety in fish, this hypothalamic circuit directly links the metabolic state of fish to their reproductive capacity. In summary, this study demonstrates the complex relationship between food intake, blood glucose regulation, and hypothalamic CCK expression. These findings provide valuable insights into the mechanisms that control appetite and metabolic responses.

The objective of this study was to investigate the mechanistic role of CCK in regulating grass carp sexual maturation through the HPG axis. Similar to mammals, sexual maturation initiation in fish is also regulated by the HPG axis, with GnRH being a major inducer of gonadotropins (GtHs) in the pituitary [11,30]. Recent research has shown that GnRH3 plays a crucial role in the proliferation and sexual differentiation of primordial germ cells during early embryonic development in zebrafish [31]. In mammals, CCK has been found to play a role in regulating GnRH expression in the hypothalamus [15,16]. In *chameleon*, some neurons show evidence of the co-expression of GnRH and CCK [32]. Additionally, recent studies in zebrafish and medaka have revealed that CCK functions as a potent FSH-RH, challenging the traditional “solo GnRH model” recognized in mammals [28,29]. In this study, both CCK and CCKR were expressed in GnRH3 neurons, and exogenous CCK could significantly induce the expression of GnRH3 in hypothalamic cells. These findings indicate that CCK may have a role in reproduction by stimulating the expression of hypothalamic GnRH.

The pituitary glycoprotein hormone gonadotropin (GTH) is a crucial regulator of vertebrate reproduction, consisting of two distinct subtypes [33]. Similar to mammals, fish pituitaries secrete two primary gonadotropins: LH and FSH. Recent research in zebrafish has revealed the crucial role of FSH and its receptor (FSHR) in female fertility and sexual maturation. The knockout of FSH or FSHR in zebrafish leads to female infertility, hindering sexual maturation [34,35,36]. These findings highlight the crucial function of pituitary-secreted FSH in regulating fish gonadal development and the initiation of sexual maturation. Identifying the key factors that govern FSH synthesis and secretion is crucial for understanding the mechanisms underlying the initiation of fish sexual maturation. In this study, we identified three CCK-Rs in grass carp: CCKRa, CCKRb, and CCKRl. Single-cell analysis revealed that *cckrb* is predominantly expressed in pituitary FSH cells. In zebrafish, the knockout of *cckbrb* significantly decreases the pituitary FSH levels [28]. Furthermore, it is important to note that the stimulatory effect of CCK on FSH production and secretion was significantly more potent than that of GnRH on FSH [30]. These results suggest that CCK is one of the main inducers of FSH in fish and exerts a direct influence on pituitary FSH cells, prompting FSH synthesis and secretion, which in turn promotes the development of fish gonads and the initiation of sexual maturation.

In addition to their well-established roles in the hypothalamus and pituitary, our recent studies have also shown that CCK and its receptors are highly expressed in the testes and ovaries of grass carp, suggesting their potential direct regulatory role in gonadal development. Similarly, the analyses of tissue expression in chickens have confirmed the expression of CCK and CCKR within gonadal tissues [37]. Furthermore, investigations have shown that CCK significantly induces the expression of gonadotropin receptors (*fshr* and *lhr*) and sex steroid hormone synthetase (*cyp19a1a*) in grass carp ovary cells. While these findings suggest a potential regulatory role for CCK in gonadal function, additional studies are needed to further elucidate the underlying mechanisms. These findings suggest that CCK can directly influence gonadal tissues, potentially exerting paracrine or autocrine effects. Similar patterns of autocrine or paracrine regulation have been demonstrated for other neuropeptides. For instance, GnRH controls zebrafish spermatogenesis as part of a complex multifactorial regulatory system [38], while gonadotropin-inhibitory hormone (GnIH) and GnRH polypeptides produced in the ovary collaboratively act in a paracrine/autocrine manner, working synergistically with gonadotropins to regulate the final maturation of zebrafish oocytes [39]. In addition, neurokinin B (NKB) induces the expression of aromatase and P450scc via the PKA/CAMKII/CREB and MEK/ERK pathways, respectively, by acting through NK3R. This directly influences gonadal development and the synthesis of sex steroid hormones in zebrafish [40]. These findings highlight the complex interaction between neuropeptides and their receptors in regulating reproductive processes and gonadal development. The article discusses the potential role of paracrine and autocrine mechanisms in regulating sex steroid hormone synthesis and reproductive function.

## 5. Conclusions

Similar to mammals, the nutritional intake of fish is a crucial factor in determining gonadal development, highlighting the close link between feeding and reproductive processes. Tissue expression analysis has shown a significant expression of CCK and CCKR within the HPG axis of grass carp. This finding strongly suggests that CCK may play a key role in regulating fish reproduction through the HPG axis. Further investigations revealed the intricate mechanisms underlying CCK’s influence at multiple levels of the HPG axis. At the hypothalamic level, CCK acts as a glucose-sensitive neuron and induces the expression of GnRH3, a critical regulator of reproductive processes, in the hypothalamus. Furthermore, at the pituitary level, CCK interacts directly with the pituitary gland, resulting in a significant increase in the synthesis and secretion of FSH in grass carp. Additionally, CCK has a direct impact on the ovary, where it stimulates the synthesis and secretion of gonadal sex steroid hormones. This is achieved by inducing the expression of gonadotropin receptors (*lhr* and *fshr*) and sex steroid synthase genes. Based on this information, it is clear that CCK plays a crucial role in regulating sexual maturation in fish. Additionally, our findings indicate that ingestion can affect gonadal development through the mediation of the satiety factor CCK, highlighting the complex relationship between nutritional intake, hormonal regulation, and reproductive processes in fish.

## Data Availability

The original contributions of this study are provided in the article and Supplementary Materials. Raw and processed data are accessible through NCBI’s Gene Expression Omnibus under the GEO series accession numbers GSE268914 and GSE269034 (https://www.ncbi.nlm.nih.gov/geo/info/linking.html, accessed on 28 November 2024). For additional information, please contact the corresponding author.

## Supplementary Materials

The following supporting information can be downloaded at: www.mdpi.com/article/10.3390/cells14020078/s1, Figure S1: Multiple alignment and evolutionary tree analysis of the CCK amino acid sequences and tissue distribution of CCKs and CCK-Rs. (A) Amino acid sequence alignment among grass carp CCK1 and CCK2. (B) Phylogenetic analysis of the CCK amino acid sequences in vertebrates. (C) Tissue expression profile of CCKs and CCK-Rs; Figure S2: Gene Ontology (GO) and Kyoto Encyclopedia of Genes and Genomes (KEGG) analysis. (A) GO classification of the assembled differential expression genes (DEGs) of grass carp pituitary cells into molecular function, biological function, cellular component. (B) KEGG pathway enrichment analysis for DEGs in the grass carp pituitary. Statistics of the top 10 enriched pathways for DEGs of up- and downregulation. Up, upregulated genes; down, downregulated genes; count, the number of DEGs; Table S1: Information for test substances in the cell culture experiments. Table S2: Antibodies used in the fluorescence immunoassay. Table S3: Markers of classical pituitary populations. Table S4: Markers of classical hypothalamus populations. Table S5: Primer sequences used for RT-qPCR.
