# Supplementary material for: Cholecystokinin (CCK) Is a Mediator Between Nutritional Intake and Gonadal Development in Teleosts
Source: Cells. 2025 Jan 8;14(2):78. doi: 10.3390/cells14020078 (PMC11763773; doi:10.3390/cells14020078)
Supplement: Supplementary file 1 [file cells-14-00078-s001.zip › cells-3369987-supplementary.pdf]

Supplementary Materials for

Cholecystokinin (CCK) is a mediator between nutritional intake and gonadal development in teleosts

Hangyu Li *et al.*

*Corresponding author. Email: zyin@ihb.ac.cn; huguangfu@mail.hzau.edu.cn

This PDF file includes:

Figures. S1 to S2
Tables S1 to S5

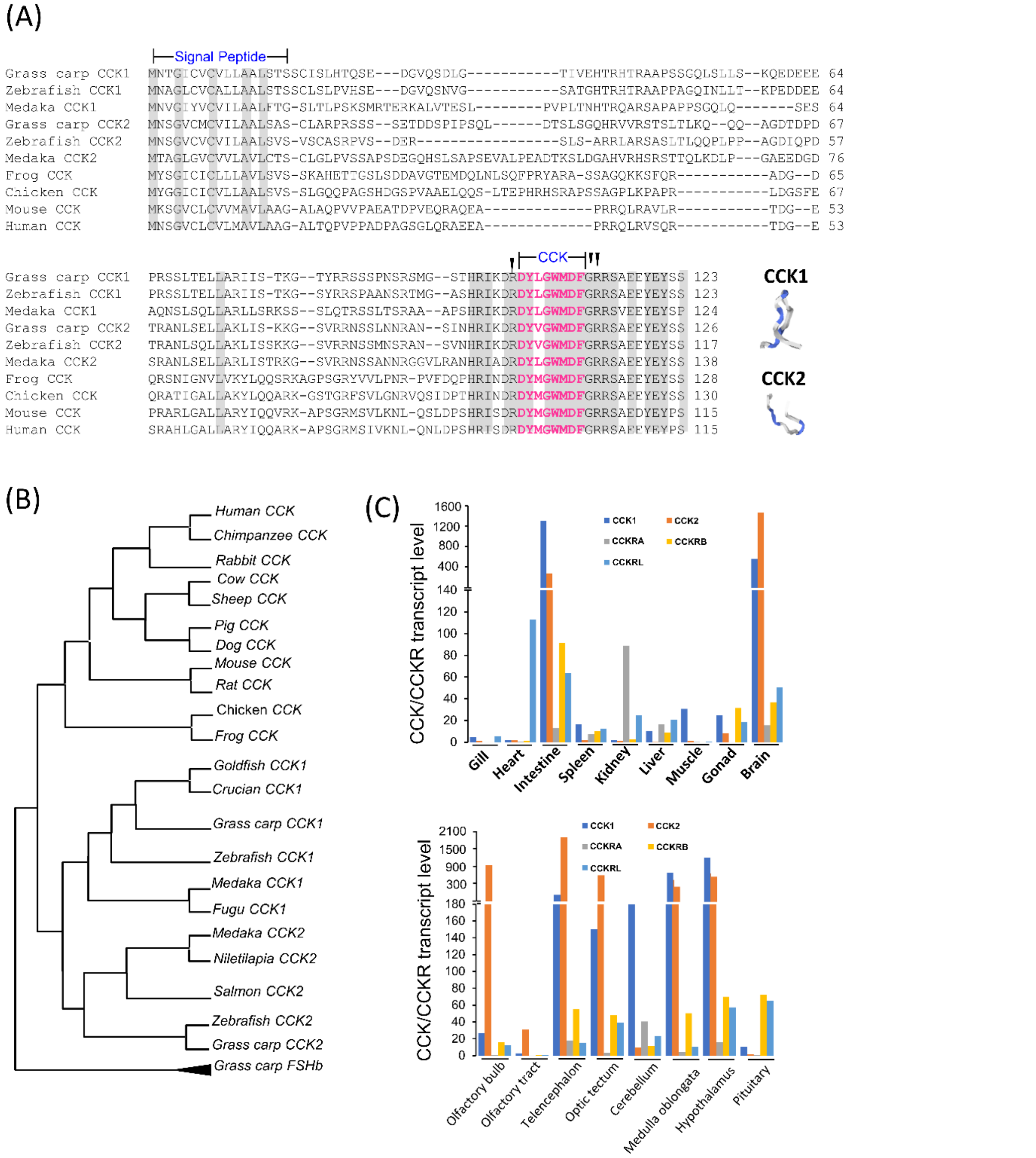

Figure. S1. Multiple alignment and evolutionary tree analysis of CCK amino acid sequences and tissue distribution of CCKs and CCK-Rs (A) Amino acid sequence alignment among grass carp CCK1 and CCK2. (B) Phylogenetic analysis of CCKs amino acid sequences in vertebrates (C) Tissue expression profile of CCKs and CCK-Rs.

(A)

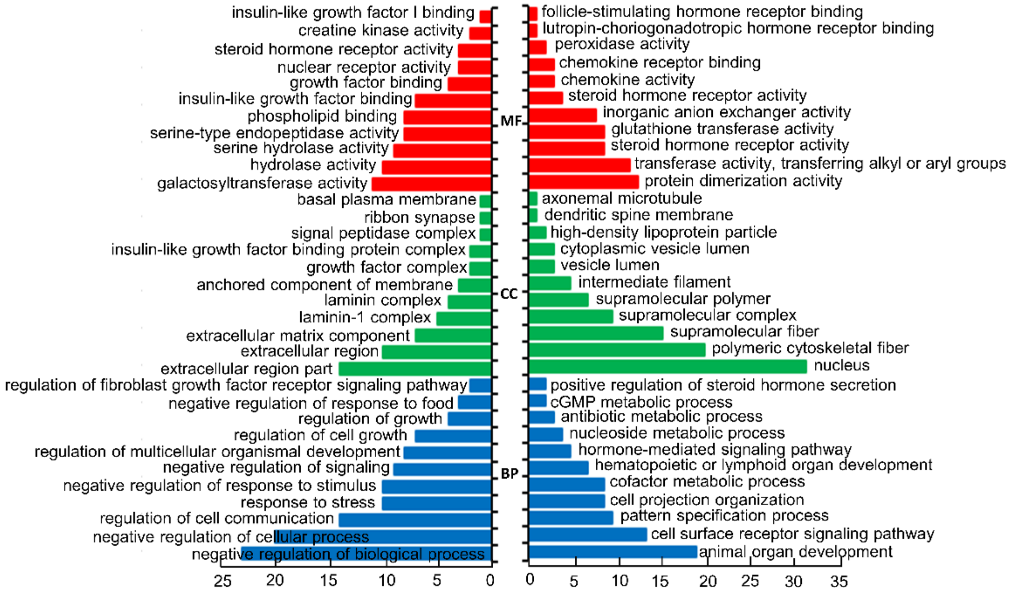

(B)

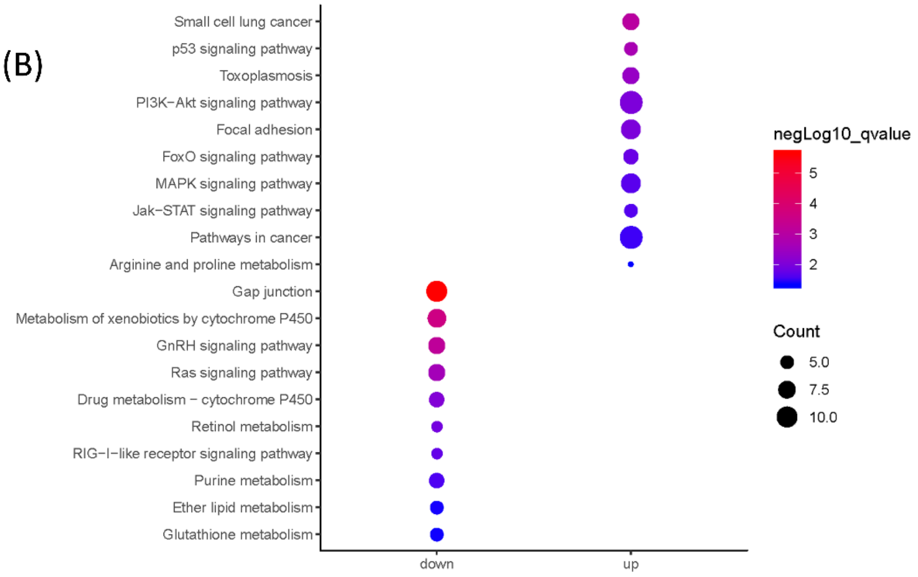

Figure. S2. Gene ontology (GO) and Kyoto Encyclopedia of Genes and Genomes (KEGG) analysis. (A) GO classification of the assembled differential expression genes (DEGs) of grass carp pituitary cells into molecular function, biological function, cellular component. (B) KEGG pathway enrichment analysis for DEGs in grass carp pituitary. Statistics of the top 10 enriched pathways for DEGs of up and down regulation. Up, up-regulated genes; down, down-regulated genes; count, the number of DEGs.

Table S1. Information for test substances in cell culture experiments

Drug name	Culture concentration	Marketplace	Product number
MDL12330A	10 µM	Sigma	M182
H89	20 µM	Calbiochem	371963
KN62	10 µM	Calbiochem	422706
2-APB	20 µM	Calbiochem	100065
U-73122	10 µM	Sigma	U6756
Nifedipine	10 µM	Sigma	U6756
GF109203X	20 µM	Sigma	B6292
Calmidazolium	1 µM	Sigma	C-100
2-Deoxy-D-glucose	300µM	MedChemExpress	HY-13966
Insulin	1 µM	MedChemExpress	HY-P73243
Glucagon	1 µM	MedChemExpress	HY-P70239

Table S2. Antibodies used in fluorescence immunoassay

Target protein	Source of antigen and sequence information	Name of Antibodies	Antibody producing species
GnRH3	recombinant grass carp GnRH3 （GenBank OP433501）	Rabbit anti-Grass carp LH antibody	Rabbit Polyclonal
GnRH3	recombinant grass carp GnRH3 （GenBank OP433501）	Mouse anti-Grasshopper LH antibody	Mouse Polyclonal
FSH	recombinant grass carp FSHβ （GenBank EF552359）	Rabbit anti-Grass carp FSH antibody	Rabbit Polyclonal
FSH	recombinant grass carp FSHβ （GenBank EF552359）	Mouse anti-Grasshopper FSH antibody	Mouse Polyclonal
LH	recombinant grass carp LHβ （GenBank EF565171）	Rabbit anti-Grass carp LH antibody	Rabbit Polyclonal
CCKs	recombinant grass carp CCK （GenBank JF912411）	Grass rabbit anti-fish CCK antibody	Rabbit Polyclonal

Table S3. Markers of Classical Pituitary Populations

Cell typs	Marker gene
Lactotropes	*prl*
Somatotropes	*gh1*
Macrophages	*c1qa, c1qb, c1qc*
Red blood cells	*prdx2*
Thyrotropes	*tshba*
Melanotropes	*pomca*
Lh cell	*lhb*
Fsh cell	*fshb*
Prop1+ group	*prop1*
Tnnt1 group	*tnn1*
Igf1 cell	*igfbp1a*
B cells	*rgs13*
Slb- gonadotropes	*slb*
Sla- gonadotropes	*sla*

Table S4. Markers of Classical Hypothalamus Populations

Cell typs	Marker gene
Red blood cells	*hbaa2, prdx2, hbba2, hbba1*
Oligodendrocyte precursor cell	*olig1*
Oligodendrocyte	*mbpa*
Ciliated cells	*arl13b*
Microglia	*apoc1*
Macrophages	*ccl19a.1，cd74a，cd74b*
Lymphatic	*mrc1a, satb1a*
Ependyma cells	*soul5*
Type I spiral ganglion neuron	*ankrd34bb*
Opioid peptide neurons cells	*pnoca*
crhbp expressing neurons cells	*crhbp*
Calicitonin neurons cells	*adma*
cck expressing neurons cells	*ccka, cckb*
Astrocyte	*gfap, glula, s100b*
Neural progenitor cell	*hmgb2a, hmgb2b*
Immature neuron	*stmn1*

Table S5. Primer sequences used for RT-qPCR

gene	Forward Primer Sequence (5'-3')	Reverse Primer Sequence (5'-3')
cck1	AACAAGAGGATGAGGAAGAACC	TGGTTGAATGGGTCTTATGAGG
cck2	CACCAGCCTTACCCTCAAACAG	AATCCATCCAGCCCACATAATCC
cckra	CTACCTCCAGCCGTCCAAGA	GGATTGACGCAGGCAGATGT
cckrb	CCTGGGTCCTGTCCTTCCTTAT	GTAGCAGCCATCTCCGTCATC
cckrl	TGTCAGCATCTCCACCTTCAGT	CGTGATGTTGTTCGGCTTGTTG
gnrh3	CATACGGTTGGCTTCCTGGT	AGGCAAACCTTTAGCGTCCA
fshβ	CAAATGCTGTTGTCTGCTT	TATGGTTCAACTACATTCCG
cyp11a	CGCTGCTATCCTGTTCAA	TGTCCACTCCTCTCAATCT
cyp17a1	TTTGACCCAGGACGATTT	TTCCCAGTCTGCTCTTAGTTTA
cyp19a1a	CGCCAGCAACTACTACAA	TTGAGAATGTCCACCTGTC
β-actin	CTGGTATCGTGATGGACTCT	AGCTCATAGCTCTTCTCCAG
